# Innovative approaches to dog-ear deformities in burn contracture release surgery: preserving skin for functional and aesthetic outcomes

**DOI:** 10.1093/jscr/rjaf360

**Published:** 2025-06-13

**Authors:** Mehdi Ayaz, Dorsa Ayaz

**Affiliations:** Burn and Wound Healing Research Center, Amiralmomenin Hospital, Surgery Department, Shiraz University of Medical Sciences, Zand Blvd. Shiraz 71339-75121, Iran; Surgery Department, Isfahan University of Medical Sciences, Hezar Jerib Avenue, Isfahan 81746-73461, Iran

**Keywords:** dog-ear deformity, burn contracture, skin grafting, reconstructive surgery, wound closure, aesthetic outcomes

## Abstract

Post-burn contractures often lead to functional impairment and cosmetic deformities. Traditional treatments involve excising tight scar tissue, which can result in dog-ear deformities. This study introduces a technique that preserves the dog-ear tissue and utilizes surplus skin to improve contracture release, enhancing both functional and aesthetic outcomes. The procedure involves dividing the dog-ear tissue into symmetrical triangular flaps, with two methods for transposing the tissue. These approaches optimize skin tension, improving contracture release without excising healthy tissue. This technique offers a promising alternative to traditional methods by preserving excess skin, reducing operative time, and improving both function and aesthetics. While not suitable for all cases, it provides enhanced options for burn contracture management. The modified dog-ear technique is an effective, versatile approach for burn contracture release, preserving skin and improving both function and appearance. Further studies are needed to assess its long-term effectiveness.

## Introduction

Post-burn contractures are a common complication resulting from the healing process of severe burns, often leading to functional impairment and cosmetic deformities [1]. These contractures occur when the scar tissue tightens, limiting movement and potentially causing permanent deformity. Standard treatment for burn contractures typically involves contracture release surgery, which aims to restore functional skin coverage by excising the tight scar tissue and using grafts or local flaps for closure [2].

During scar revision or excision procedures, the formation of a dog-ear deformity is a potential complication. A dog ear is a dome-shaped irregularity that results from the removal of skin, often leading to an undesirable excess of tissue i.e. cosmetically unacceptable to the patient. Traditional management typically involves excising the dog ear along with the surrounding excess skin [3].

In this study, we present an innovative technique that not only avoids the excision of the dog ear but also utilizes the surplus skin to release the surrounding contracture, thereby improving both functional and aesthetic outcomes.

## Materials and methods

This study includes a retrospective case series of 15 female patients, aged 20–45 years, who sustained burns involving more than 20% total body surface area (TBSA). The contractures were located on the abdomen, upper, and lower extremities, and all surgeries were performed ~2 years after the initial burn injury for contracture release.

There was no strict preference between the two procedures. Procedure 1 was used in 10 patients, while Procedure 2 was performed in 5 patients. The main consideration for choosing Procedure 2 was the necessity of more flap incisions to accommodate greater tissue redundancy and reduce the risk of flap necrosis. The larger flap size in Procedure 2 required more extensive incisions but was essential for achieving optimal outcomes and feasibility, given the nature of the contractures. The choice between procedures was made intraoperatively, based on flap characteristics and the extent of tissue involved.

Written informed consent was obtained from all patients prior to surgery. Additionally, consent for the publication of their photographic images was obtained. Due to the retrospective and illustrative nature of this surgical innovation report, institutional ethical approval was not required.

### Technique

#### Step-by-step description

**Figure 1:**
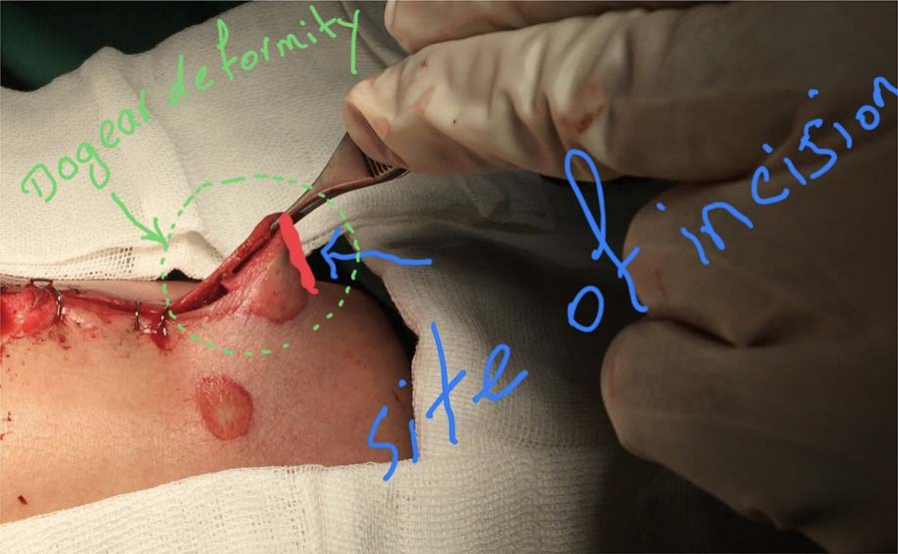
The initial incision site was marked at the apex of the dog-ear deformity.

1) **Identification of the Contracture and Dog-Ear Deformities**:
  - During the scar contracture release, identify the dog-ear deformities at the surgical wound’s edges.
2) **Design of Triangular Flaps**:
  - Mark the skin excess forming the dog-ear deformities and plan their conversion into triangular flaps. The flaps’ size and orientation depend on the contracture’s location and direction (Fig. 1).
3) **Flap Formation**:
  - Carefully excise the scar tissue causing the contracture.
  - Elevate the triangular flaps from the dog-ear deformities, ensuring adequate vascularity.
4) **Flap Advancement and Anchoring**:
  - Advance the flaps to cover the released area of the contracture.
  - Secure the flaps with absorbable sutures, ensuring proper alignment and tension.
5) **Closure**:
  - Close the remaining wound edges with non-absorbable sutures. Adjust the flap positioning as needed for optimal aesthetic and functional outcomes.

This technique not only avoids discarding viable skin but also transforms it into a functional and aesthetic reconstructive tool.

#### Detailed steps of the technique

**Figure 2:**
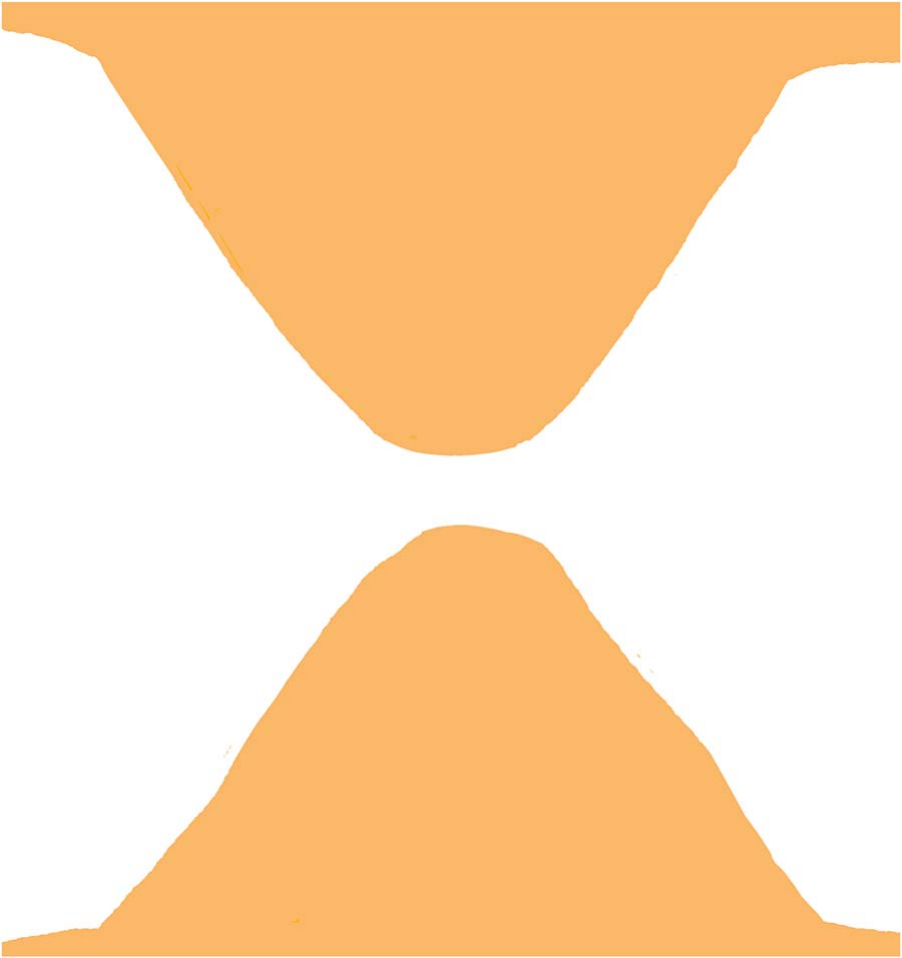
Bilateral triangular flaps formed during dog-ear correction, each with a base attached to the surrounding skin on either side of the wound.

In this technique, we begin by making an incision on the dome of the dog ear to divide it into two similar parts on both sides (Fig. 1). These newly formed parts of the dog ear are identical on both sides of the wound and are triangular, with only their base attached to the surrounding skin on both sides of the dog ear (Fig. 2). We have two choices for the next step.

**Figure 3:**
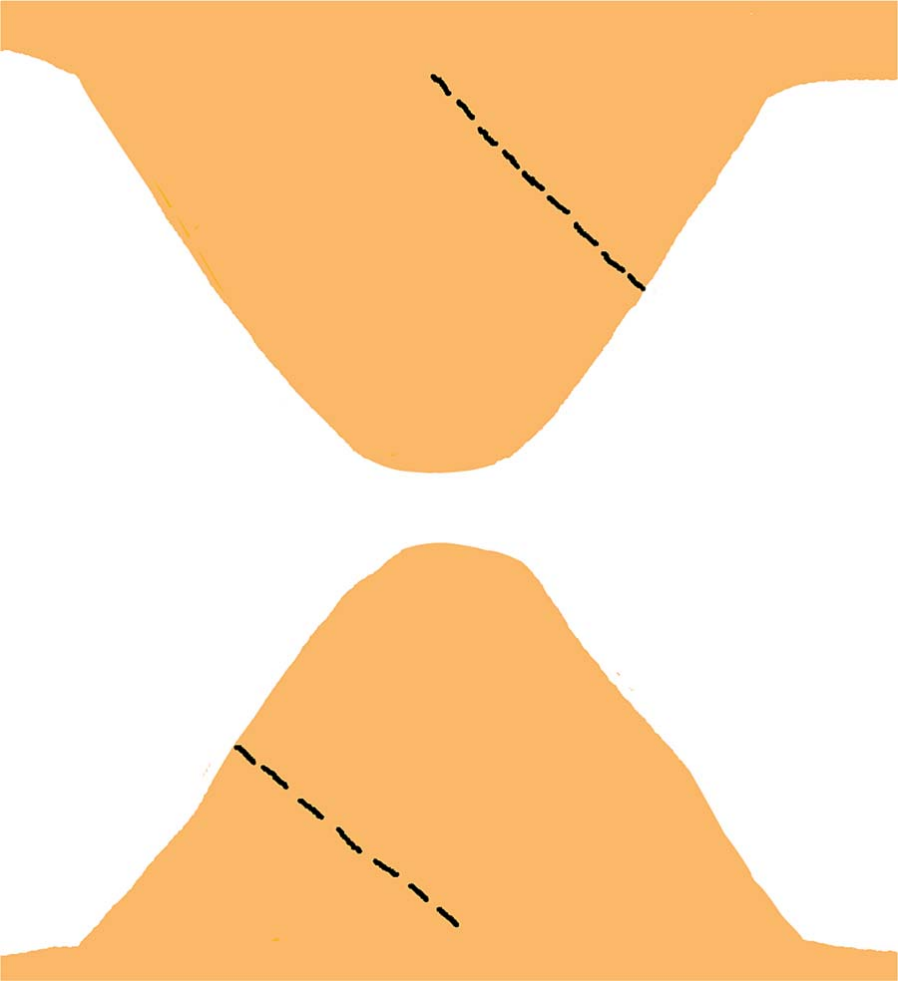
Incisions are made from the midpoint of the lateral edge to the midpoint of the base on one triangular flap, mirrored on the contralateral triangular flap to facilitate symmetrical reconstruction.

**Figure 4:**
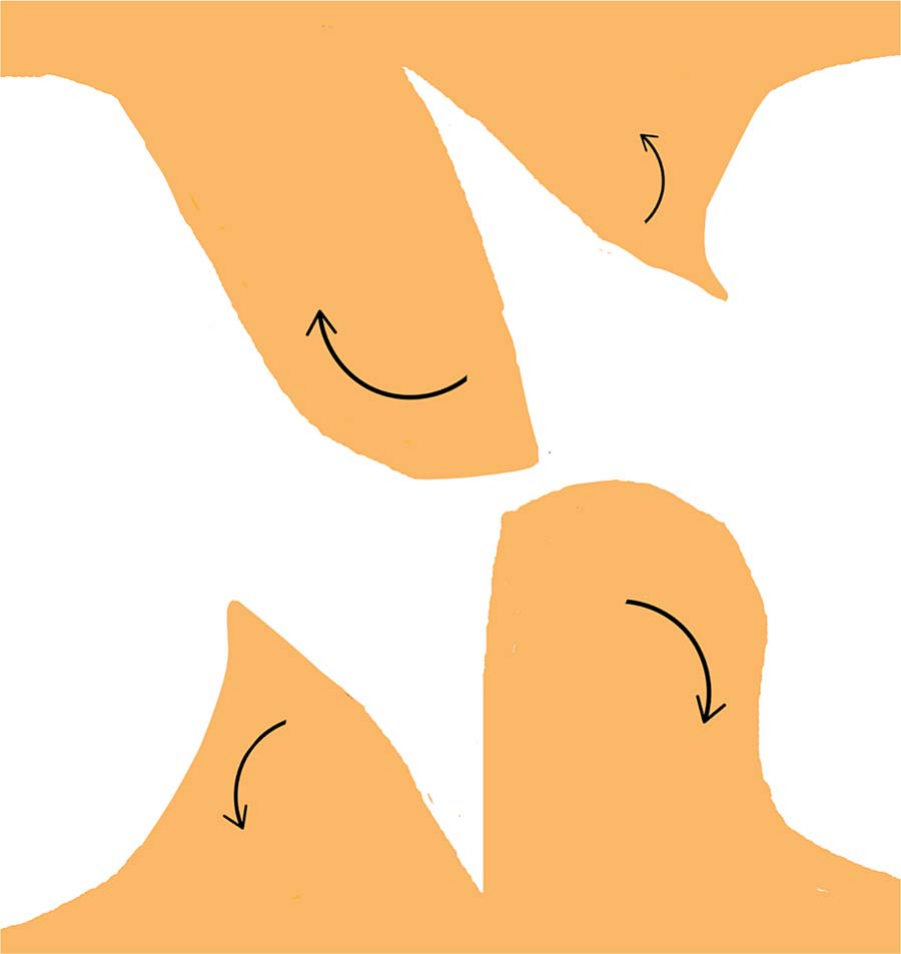
Retraction of the incised edges to widen the opening, creating adequate space for interlocking the contralateral flap without excessive tension.

**Figure 5:**
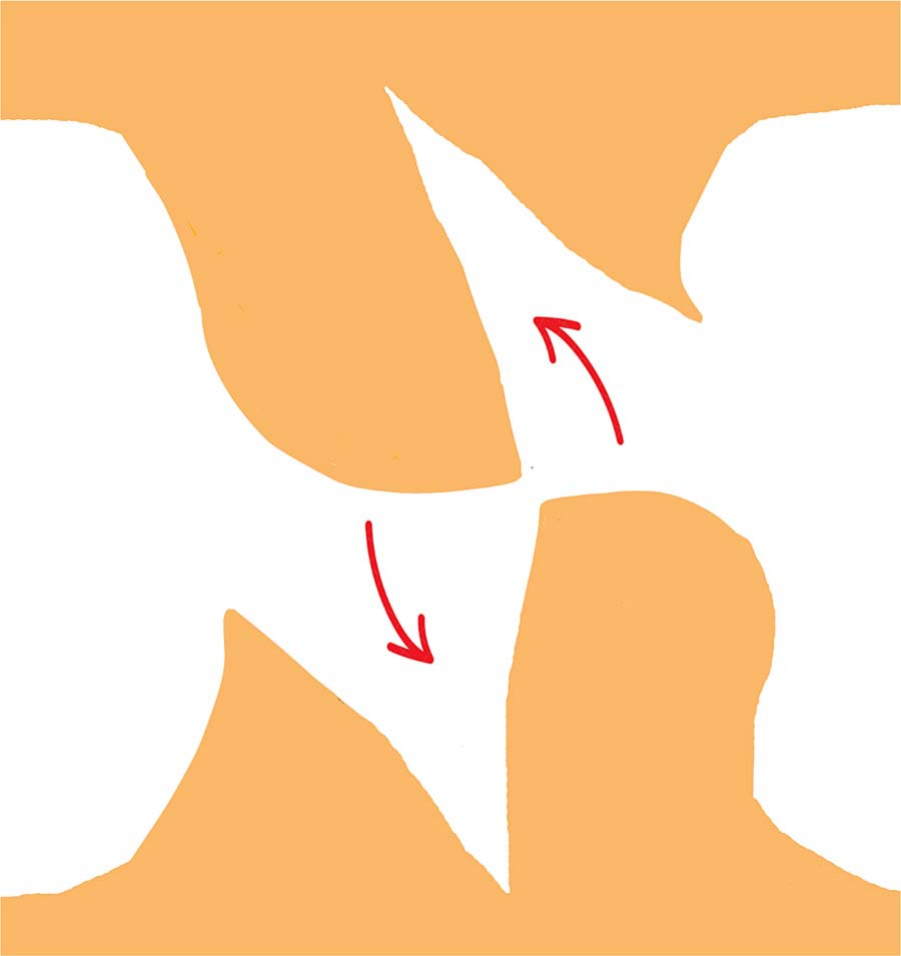
Transposition of the arrow-shaped portion of each flap into the contralateral wedge defect with slight rotation, ensuring optimal contour, tension distribution, and preservation of vascular integrity.

**Figure 6:**
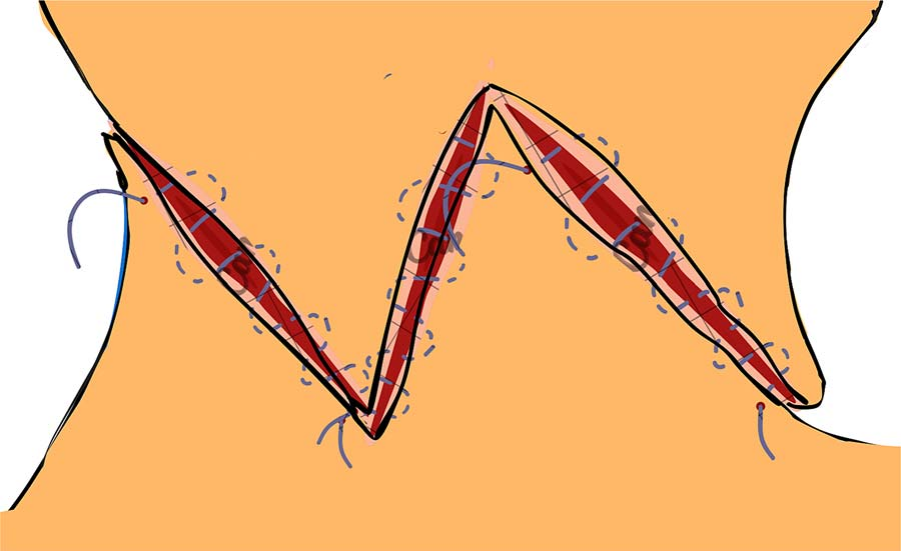
Final positioning and fixation of the flaps using non-absorbable sutures. Interrupted or horizontal mattress sutures are placed along the wound margins to ensure proper alignment, reduce tension, and enhance both functional and aesthetic outcomes.

**Figure 7:**
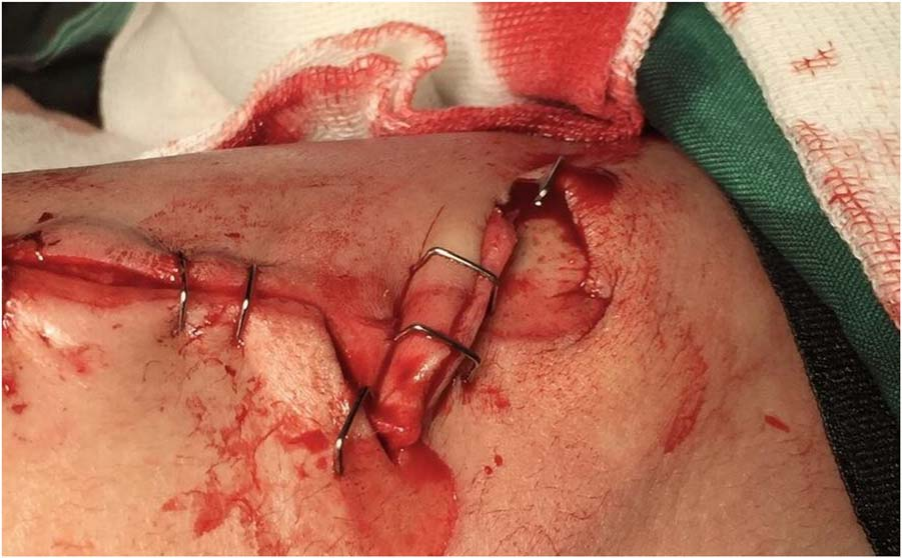
Postoperative clinical image showing the final positioning and suturing of the flap in an actual patient. Image used with written informed consent. Identifying details have been omitted to protect patient confidentiality.

**Figure 8:**
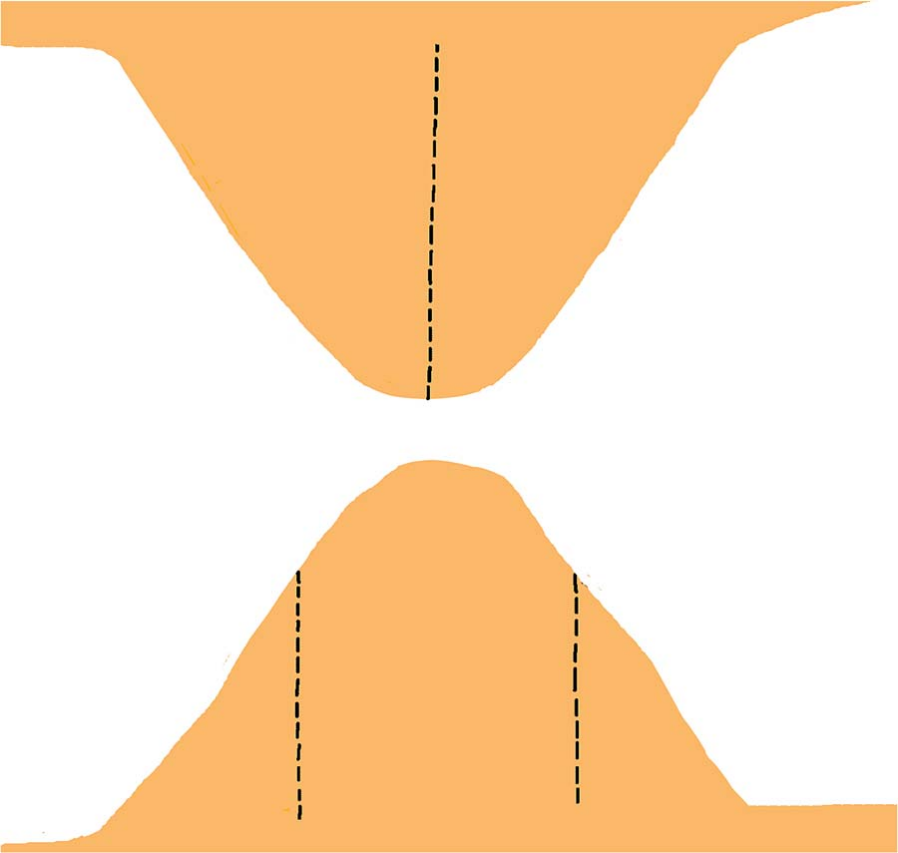
Vertical incision made from the apex to the base on one flap, and two identical incisions on the contralateral flap, positioned one-third from the apex along the lateral edges, extending straight and parallel to the triangle’s altitude down to the base.

**Figure 9:**
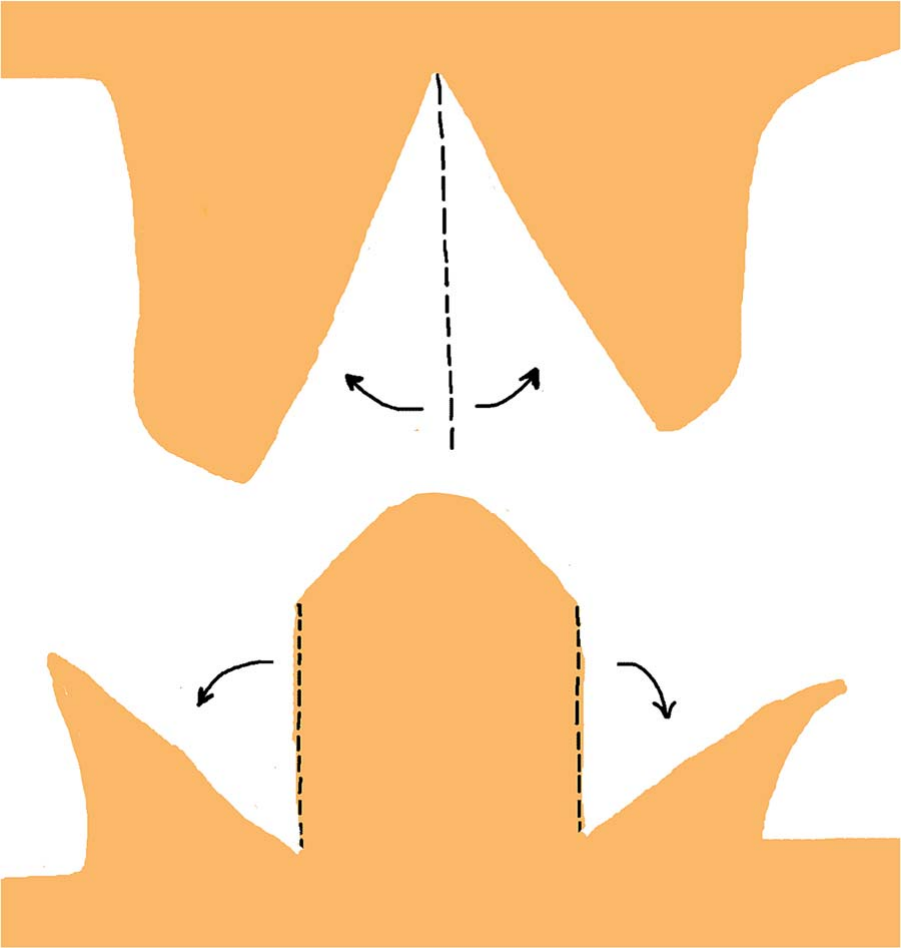
Retraction of the wedge defects to allow precise fitting of the arrow-shaped prominences into their corresponding defects, ensuring proper alignment without excessive tension.

**Figure 10:**
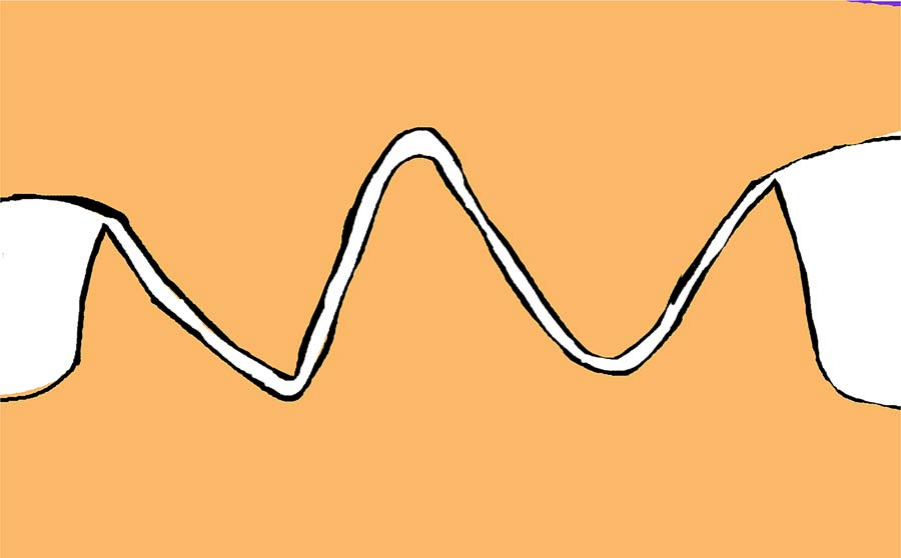
Properly aligned tissue edges interlock securely, ensuring a stable, tension-free closure that preserves both functional and aesthetic outcomes.

1) **First Option:**

- On one triangular flap, make an incision from the lateral edge’s midpoint to the base’s midpoint. Repeat this on the opposite lateral edge of the contralateral triangular flap (Fig. 3).
- Gently retract the incised edges to widen the opening, creating sufficient space to accommodate the corresponding tissue from the contralateral flap. Ensure the gap allows precise interlocking of the flaps without excessive tension (Fig. 4).
- With slight rotation, transpose the arrow-shaped portion of one flap into the wedge defect of the contralateral flap and vice versa, ensuring optimal contouring, tension distribution, and vascular integrity (Fig. 5).
- Once the flaps are correctly positioned, secure them using non-absorbable sutures. Place interrupted or horizontal mattress sutures along the wound margins to maintain proper alignment, minimize tension, and optimize both functional and aesthetic outcomes (Figs 6 and 7).

2) **Second Option:**

- As illustrated in Fig. 8, make a vertical incision from the triangle’s apex to the base on one flap. On the contralateral flap, create two identical incisions along the lateral sides. These incisions should be positioned approximately one-third of the way from the apex, along the edge of the lateral sides, extending straight and parallel to the triangle’s altitude down to the base of the triangle.
- This results in two wedge-shaped defects, bordered by arrow-shaped prominences on both sides of one flap, and a larger central wedge defect on the contralateral flap, with arrow-shaped prominences on each side.
- Retract the edges of the wedge defects to allow the arrow-shaped prominences to fit precisely into their corresponding defects, ensuring no undue tension (Fig. 9).
- When the tissue edges are properly aligned, they will interlock securely, allowing for a stable, tension-free closure that maintains both functional and aesthetic outcomes (Fig. 10).

This procedure provides the opportunity to have more skin available for improved contracture relief. Additionally, this technique can help to overcome stiffness in the surrounding scarred skin. It also resolves a dog ear without requiring skin excision, which is sometimes normal and non-scarred. However, in a patient without any contracture or skin deformities around a dog ear, it is better to excise it to avoid elongating the operation time.

This technique provides surgeons with more choices for reconstructing scars and correcting contractures.

The damaged appearance of skin edges in our figures results from residual scar tissue rather than intraoperative trauma.

## Results

This technique was applied to 15 female patients aged 20–45 years, each with a history of deep second- or third-degree burns involving over 20% of TBSA. All patients underwent contracture release surgery at least 2 years after the initial burn injury. The contractures were located on the abdomen, upper, or lower extremities.

Procedure 1 was performed in 10 patients. Procedure 2 was used in 5 patients, selected for cases where the flap size was larger and more extensive incisions were necessary to allow for tension-free closure and reduce the risk of tip necrosis.

All surgeries were successfully completed. No postoperative complications such as flap necrosis, infection, wound dehiscence, or recurrence of contracture were observed in any patient during a 6-month follow-up period.

## Discussion

Post-burn contractures pose significant challenges in reconstructive surgery, often necessitating excision and grafting to restore function [1]. However, traditional methods may lead to dog-ear deformities, requiring further excision and potential loss of viable skin [2, 3]. This study presents an innovative technique that preserves excess skin, utilizing it to enhance contracture release and improve both functional and aesthetic outcomes.

By strategically dividing and transposing dog-ear tissue, this technique optimizes tissue preservation and redistributes skin tension, enhancing contracture relief, particularly in cases of extensive scarring. Additionally, avoiding unnecessary excision reduces operative time, minimizes closure tension, and enhances aesthetic outcomes, improving patient satisfaction [3].

Option 2 of our technique closely resembles the double-opposing Z-plasty approach, which is traditionally used to redistribute tension and lengthen contracted tissue. We have added a reference to this method to highlight the conceptual foundation shared between our technique and this established reconstructive strategy [4].

Additionally, it shares similarities with the ‘jumping man flap,’ a modification of the double-opposing Z-plasty used in specific contracture releases, such as in syndactyly or periorbital areas [5]. In total, 15 adult female patients between the ages of 20 and 45 underwent surgery using this technique. All patients had experienced second- or third-degree burns affecting over 20% of their TBSA, and the procedures were performed at least 2 years after the initial burn injury. Procedure 1 was used in 10 cases, while Procedure 2 was applied in 5 patients with larger dog-ear flaps, where extended incision design was necessary to ensure perfusion and feasibility. The only determining factor in selecting between the two techniques was flap size and design, with no strict preference. No postoperative complications, including tip necrosis, infection, dehiscence, or recurrence, were observed during the 6-month follow-up period. Careful intraoperative planning and assessment of perfusion were essential in minimizing risk and ensuring flap viability. Moreover, in choosing between the two techniques, the clinical judgement of the operating surgeon played a crucial role in minimizing complications such as tip necrosis. Individual assessment of tissue quality, vascularity, and anatomic location was essential in guiding the selection of the most appropriate approach.

Despite its advantages, this approach may not be suitable for all cases. In very small dog-ear deformities—especially in anatomically delicate areas such as the fingers—the technique may increase the risk of tip necrosis due to limited vascular supply. Therefore, cautious case selection is critical. Standard excision remains preferable in the absence of significant contractures or when the available tissue is insufficient.

Furthermore, the decision to use Option 1 or Option 2 depends on both the size and orientation of the deformity. Option 2 is generally favoured for larger, more elongated dog ears, while Option 1 is typically selected for moderate-sized, symmetrical cases. Intraoperative assessment and surgeon preference also influence this choice.

Precise execution is essential, highlighting the need for surgical expertise.

In conclusion, this technique expands reconstructive options by preserving and repurposing excess skin, offering a promising alternative for managing post-burn contractures. However, additional clinical studies, including prospective trials, are needed to validate its safety and long-term efficacy.

## Conclusion

In conclusion, the modified dog-ear technique is a useful tool for plastic and burn surgeons to correct contractures while preserving newly formed skin remnants. By utilizing excess skin from the dog ear, we can achieve better cosmetic results and improve the function of the affected area. This approach is especially beneficial in cases where multiple releases are required or when there is a lack of surrounding skin for local flap procedures. The Z-plasty or local flap technique allows us to redistribute tension along the incision line and prevent further contracture formation. Overall, this technique provides an effective solution for correcting contractures and improving the function and appearance of the affected area.

The modified dog-ear technique is a useful tool in the armamentarium of plastic surgeons for contracture release in various anatomical locations. Its versatility, simplicity, and effectiveness make it a valuable option for patients with contractures.
